# On a New Mode of Operating for Fistula in Ano

**Published:** 1884-05

**Authors:** Edward W. Jenks

**Affiliations:** Chicago


### Selections.

---

### ON A NEW MODE OF OPERATING FOR FISTULA IN ANO. By EDWARD W. JENKS, M.D., LL.D., Chicago.

Although diseases of the rectum and anus do not belong exclusively to the domain of gynecology, they are of such common occurrence among women, and are so frequently encountered by the gynecologist, either associated with other pelvic disorders of women or as the only disease of that region, that the writer offers no apology for presenting to the Society a brief paper with the foregoing title.

It may not be the part of wisdom to entitle any mode of operating or plan of treatment as new, in view of the fact that the history of medicine shows that much which we term new was known hundreds of years ago. I have, however, ventured to designate as new the operation described in this paper, for the reason that I find nothing analogous to it described in modern works.

In all literature on the subject of fistula in ano, including the latest systematic treatises on rectal and anal diseases, may be found frequent allusions to incontinence of feces and flatus following, and consequent upon, surgical operations in the anal region. As this is dependent on the condition of the sphincter muscles, it differs greatly in degree, from slight weakening to total loss of contractile power—*i. e.*, partial or complete incontinence. It is not uncommon for partial incontinence to succeed an operation for fistula in ano when done by skilled hands and after the most approved method of the present time. Allingham says: "In operating upon women suffering from fistulæ, cut as little as possible, for anything like too free incisions are apt to end in incontinence of feces, or, at all events, in such partial loss of power in the sphincter as to prevent the patient retaining flatus, a result which, I need scarcely say, is a most disagreeable one." "A patient who suffers from inability to retain flatus or feces is in a most unpleasant condition ; in fact, some sensitive persons would not undergo any operation which was at all likely to induce such a state, and would prefer any physical suffering rather than the perpetual fear of being in any way offensive to others." A surgeon of distinction, who has given special attention to diseases of the rectum, recently informed the writer that among the many anal fistulæ he has incised "the number is few in which the function of the sphincters is as perfectly performed as it was previous to operating." I understood by his remark that, while there was not what he designated as actual incontinence in any degree in the majority of his cases, there was less control of liquid feces and flatus than existed before the sphincters were incised.

A little over a year ago I was consulted by a woman on account of rectal incontinence. This condition was caused by an operation for fistula which scarcely involved a single fiber of the upper sphincter. The operator in this case was not an experienced or distinguished surgeon, this being his first operation about the rectum. The causes of incontinence, in my opinion, were: (*a*) a perineum previously weakened by being torn in childbirth, and (*b*) the cutting of the lower sphincter obliquely.*

Two cases have quite recently been brought to my notice of complete incontinence succeeding operations for fistulæ. Both of these women were operated upon by gentlemen of experience and acknowledged skill. In each the internal sphincters were cut, but not entirely through. One of these women, since her operation for the fistula, has been subjected to two additional operations for the incontinence, without deriving any benefit therefrom.

A suit against a surgeon for malpractice which is now pending

* My own observation has convinced me that Allingham has not overestimated the importance of incising the sphincters at right angles to their muscular fibers, and that even an oblique incision of the lower sphincter may result in complete incontinence.

in one of the courts of a Western city, involves the subject under consideration, and is of sufficient interest to deserve more than a mere allusion. One trial, resulting in a disagreement of the jury, has been held, and another, which has been ordered, will soon occur. In this instance suit has been brought by a woman against a surgeon of prominence, in which she claims damages to the amount of several thousand dollars on account of the unskilful and improper performance of an operation for fistula in ano upon her own person by the defendant in the case. She states that, although she suffered inconvenience and discomfort prior to the operation for the fistula, she is now, by reason of fecal incontinence, in a much worse condition. She has made the suit appear still stronger in her favor, as I have been informed, by testifying that she has undergone an additional surgical operation for the purpose of curing the incontinence, but has derived no benefit therefrom.

From all I can learn relating to the treatment of this patient, the surgical operation was performed as it is generally done by surgeons, and after the manner advised by the majority of modern authorities. My friend and former pupil, Professor H. O. Walker, of Detroit, was an expert witness in this suit, and while in conversation with him concerning it, and of the various modes of treating different forms of anal fistulæ, it occurred to me that some cases might be successfully operated on by a method similar to the operation for a torn perinæum. By so operating, it seemed both to Dr. Walker and myself, that immediate union would be secured, a tedious convalescence avoided, and incontinence rendered impossible.

I have not had the opportunity of performing the operation which I have designated as a new one for fistula as many times as I would like before bringing it to the notice of the Society, but my limited experience has led me to fully believe that it is an improvement upon the method of incising and expecting the gap to be healed by granulation, and also that it is of sufficient importance to merit a trial at the hands of other surgeons. I have had but two patients upon whom I have tried this operation, and with each the success was all that could be desired.

The mode of operating as I have performed it may be briefly described as follows: First, after determining the routes of the fistulous tracts, they are incised after the usual method, aiming, in every instance where the incision involves either sphincter muscle, to have the incision at right angles to the muscular fibers. The next step is to carefully dissect out the so-called pyogenic membrane, or all of the lardaceous tissue and the cartilaginous substance along the route of the fistulæ. This can be done with curved scissors or a cutting curette. It is not unusual to find several bleeding vessels in the fistulous tract; these should be secured by torsion in preference to ligatures, but if ligatures become a necessity, then Chinese silk is to be preferred. The surgeon frequently finds, after cutting a fistula, that there are, overlapping the incision, portions of thin livid skin of low vitality. This should be entirely cut away in all instances, but in the operation under consideration the edges of the incised skin should be pared until they can be brought together perfectly, and then, by deep sutures, the incised parts maintained in perfect apposition until adhesion is effected. The deep sutures can be adjusted in the easiest manner by a Peaslee's needle or the common perineal needle, and should be buried beneath the bottom of the incised fistulous tract, so that no portion is visible except the two ends protruding from the integument. In addition to deep sutures, it is in most instances necessary to insert superficial sutures alternately with the deep ones, that the strain upon the latter may be lessened and the edges kept in apposition. The severed portions of the sphincters are united by sutures in the same manner as is done in complete laceration of the perinæum, for the knowledge of which we are indebted to our distinguished Fellow, Dr. Emmet. If an operation for anal fistula by the method advocated here proves a success, it is mainly owing to the severed ends of the sphincters having been held in perfect apposition a sufficient length of time to effect their union. For this purpose silver-wire sutures properly adjusted are, without question, preferable to silk. I have used both silk and silver with equally good results, but prefer the latter, deeming it the most reliable if the operator has been accustomed to its use in surgical work.

If an opening into the rectum of the tract of the fistula is of such a height that its incision includes any portion of the internal sphincter, it is necessary to use additional sutures within the rectum to bring and maintain in apposition such incised parts as the external sutures will fail to do.

I have used for this purpose Chinese silk, for the reason that, while sutures of this material are commonly of sufficient strength to hold the parts together until union is secured, they do not require removal by the surgeon, as they are discharged in a few days. If it is deemed desirable for any reason to use stronger sutures, the best material is "ironized silk" * (1 to 3), because of its black color, which renders it much easier than white silk to remove from the rectum. In case of there being several fistulous openings in the rectum, an attempt to cure all of them at one operation by this new method would doubtless result in failure. The same is true of the ordinary mode of operating, as is well known to all who have had much experience in rectal surgery. With several incisions left to heal by granulation when the sphincters are included, incontinence is quite sure to follow. We are advised, by those who are entitled to speak authoritatively on the subject, that for this reason an attempt should not be made to cure all by a single operation. In the mode of operating which is advocated in this paper, more sinuses can be safely cut than by the old method, and yet too much should not be attempted in one operation. Very much depends on the character of the fistulæ; as for instance, if there is anything like the ulceration common with consumptives at the rectal end of a sinus, an attempt should not be made to bring the rectal edges together by sutures; yet some of the other sinuses can be incised and be made to unite by sutures; nor should any fistula be incised if it is acutely inflamed. In either one of these conditions pus would be very likely to burrow and new sinuses be formed. Allingham gives this as a reason why we "should never operate on a fistula that is from any cause acutely inflamed."

The route of an internal fistula may be straight or tortuous, and, if either, sometimes it will run close to the mucous mem-

---

* I am indebted to Prof. William H. Pancoast, of Philadelphia, for suggesting the use of ironized silk in the operation, and its advantage in this and other operations pertaining to gynæcological surgery.

brane and have its rectal opening above the internal sphincter, or it may run behind both sphincters and open above the internal, or, while extending a distance above the internal sphincter, as a probe indicates, its rectal opening may be quite a distance below its upper limit. If such fistulæ are incised their entire length, and then left to heal by granulation, incontinence in some degree is inevitable. If some of these fistulæ are not too deep, or do not communicate with abscesses, they may be cured by the method heretofore described. In the absence of exact knowledge concerning the use of sutures, in case all the fibers of both sphincters have been cut through, I deem it the best and most prudent course to pursue, when such incisions seem to be required, to insert an elastic ligature at the upper limit of the fistulous tract, and after causing it to cut through the internal sphincter at right angles to its muscular fibers, the remaining portion may be incised and united by sutures in the manner already described, or left to be cut entirely through by the elastic ligature, as in the judgment of the surgeon may seem best in each case.

It is as impossible to follow any exact rules as it is needless to give them for the surgical treatment of fistulæ; especially is this true of many internal fistulæ. While, in my opinion, there are some fistulæ in which sutures are better than any other mode of treatment, others can be more certainly cured by means of the elastic ligature; then, again, there are doubtless some which can only be treated by being incised and left to heal by granulation.

As regards the manner of adjusting sutures, the same exact rules cannot be followed as in perineorrhaphy, or other plastic operations about the pelvic organs, for the reason that there is such a lack of uniformity in the shape and location of fistulæ. For the purpose of obtaining success in the operation under consideration, adherence to the general rules of surgery, the teachings of experience, and the ingenuity of the operator are requisite in each individual case.

Having described the mode of operating in anal fistulæ, and endeavored to show its advantages, I will further illustrate this method by an account of cases operated on:

The first patient was an inmate of my private hospital, Mrs. A., age 25; nulliparous, of a strumous diathesis. The fistulæ had troubled her four years. She had two distinct fistulæ, opening into the rectum half an inch apart; one fistulous tract was straight, having its external opening in the direction of the tuber ischii, opening on the left about two inches from the anus, while the internal was immediately below the internal sphincter. The other fistula had its tract above the first, and its external opening in the fleshy part of the left buttock, three inches and a half from the anus. There was, in addition, a lateral sinus in the form of a blind fistula leading from this one, and situated about midway between the rectal and external orifice, and directed upward. The first sinus was incised, packed with marine lint, and allowed to heal by granulation. The second one, with its lateral branch, was treated after the method advocated in this paper. As this was an experimental operation, I was slow in carrying out the details. The tract of the fistula was carefully cleared of all adventitious tissue by means of small curved scissors, the edges and deeper parts trimmed, and bleeding vessels secured by torsion, as has been previously described; then sutures were inserted so that no part was visible in the wound. This was done with great care beneath the tract of the main sinus and its lateral branch. The severed sphincter was brought together by sutures in a manner similar to Emmet's operation for complete laceration of the perinæum. It could then be seen that within the rectum there was a gap that needed to be closed, or else the fistula would remain unhealed. Four interrupted sutures of Chinese silk were used within the rectum for this purpose.

Nine days later, after a thorough evacuation of the bowels, all sutures excepting those of Chinese silk were removed; union was complete the entire length of each sinus. The other sinus, which had been simply incised and packed with marine lint, required a continuation of similar treatment for over a month before the gap had healed by granulation. This one might have been cured in the same time as the others if it had been treated in the same manner, as it was a more favorable case for the use of sutures than the others; but, instead of being healed in ten days, as the others were, it kept the patient confined to her room for weeks, requiring daily dressings and causing discomfort that might have been avoided.

The second case was in private practice, the subject being Mrs. B., age 32; a widow. She had borne two children, and her perinæum was torn to the external sphincter. The fistula had existed for six years, and was attributed by her to a difficult instrumental labor from which she was seriously ill several weeks. The external opening of the fistula was on the left side, two inches and a half from the anus; the route of the fistulous tract was quite straight through one edge of the perinæum to its rectal extremity in the recto-vaginal septum, at the lower margin of the internal sphincter. The appearance of the fistula after being incised was similar to that of a large-sized artery. The dense cartilaginous substance which caused this appearance was carefully dissected out by means of small, sharp-pointed scissors.

There were no pockets for the retention of pus, no lateral or crooked sinuses leading into it; but, after being cut, and all the livid skin was trimmed away from the edges, its appearance was similar to a recent incision which would readily heal if it was properly brought together, and so maintained for a sufficient length of time. This was done by means of sutures inserted in the same manner as in the first case. Within the rectum three interrupted sutures of Chinese silk were introduced. Convalescence was rapid; the bowels were moved by means of laxatives and an enema on the ninth day. On the tenth day all external sutures were removed, two of the rectal sutures had disappeared, and no attention was paid to the remaining one. Union of all portions of the late fistula seemed to be perfect, and a cure was effected.

In concluding this paper, there are a few points to which I desire to direct attention:

1. In my own opinion, while this mode of operating is not adapted to *every* form and variety of anal fistulæ, I believe it by far the best method in the majority of cases.

2. The long and tedious convalesence inseparably connected with the ordinary mode of operating is by this mode avoided.

3. Other advantages of this mode of operating are (*a*) securing nion by the first intention, and (*b*) the prevention of incontinence.

4. In cases where the external sphincter has become rigid, it is not easy to hold its incised ends in close apposition without forcibly dilating it with the thumbs prior to operating, or else by an incision opposite the tract of the fistula of sufficient depth to temporarily paralyze the muscle. In this way the sutures in the sphincter will render better service.

5. For the purpose of facilitating the introduction of sutures within the rectum, there are two means, as follows: (*a*) the introduction into the rectum of a Sim's speculum; the eversion of the lower part of the rectum by means of one or more fingers being introduced into the vagina, and then turning out a portion of the rectum through the external sphincter.

